# Encephalitis and cytokine storm secondary to respiratory viruses in children: Two case reports

**DOI:** 10.3389/fped.2022.1049724

**Published:** 2023-01-20

**Authors:** Pollyana C. P. Santos, Adrian J. Holloway, Jason W. Custer, Tomaz Alves, Liliana Simon

**Affiliations:** ^1^ Pediatric Critical Care Observation Program, Department of Pediatric Critical Care Medicine, University of Maryland Medical Center, Baltimore, MD, United States; ^2^ Department of Pediatric Critical Care Medicine, University of Maryland Medical Center, Baltimore, MD, United States; ^3^ Division of Comprehensive Oral Health, Adams School of Dentistry, The University of North Carolina at Chapel Hill, Chapel Hill, NC, United States

**Keywords:** encephalitis (MeSH), cytokine storm, children, respiratory syncytial virus, parainfluenza virus

## Abstract

**Introduction:**

Encephalitis is a syndrome characterized by brain damage secondary to an inflammatory process that is manifested by cognitive impairment and altered cerebral spinal fluid analysis; it may evolve with seizures and coma. Despite viral infections representing the main cause of encephalitis in children, respiratory syncytial virus (RSV) and parainfluenza virus are mostly associated with respiratory presentations. Uncommonly, the inflammatory phenomena from encephalitis secondary to viral agents may present with an exacerbated host response, the so-called cytokine storm. The link between these infectious agents and neurologic syndromes resulting in a cytokine storm is rare, and the underlying pathophysiology is still poorly understood.

**Case presentation:**

A 5-year-old girl and a 2-year-old boy infected with parainfluenza and RSV, respectively, were identified through nasopharyngeal polymerase chain reaction. They were admitted into the pediatric intensive care unit due to encephalitis and multiple organ dysfunction manifested with seizures and hemodynamic instability. Magnetic resonance imaging findings from the first patient revealed a bilateral hypersignal on fluid-attenuated inversion recovery in the cerebral hemispheres, especially in the posterior parietal and occipital regions. The girl also had elevated IL-6 levels during the acute phase and evolved with a fast recovery of the clinical presentations. The second patient progressed with general systemic complications followed by cerebral edema and death.

**Conclusion:**

Encephalitis secondary to respiratory viral infection might evolve with cytokine storm and multiorgan inflammatory response in children.

## Introduction

Encephalitis is a central nervous system (CNS) inflammatory condition manifested with fever, impaired mental status, seizures, altered cerebral spinal fluid (CSF) testing, and abnormal findings on magnetic resonance imaging (MRI) (1). Viral infections are the main cause of this disease, accounting for more than 50% of all encephalitis etiology (2). Despite respiratory syncytial virus (RSV) and human parainfluenza virus (HPIV) presenting well-recognized respiratory manifestations, they are rarely reported as causative agents of encephalitis (2, 3). Moreover, the pathophysiological mechanisms for brain damage and systemic inflammatory status secondary to infection by these viruses are not fully understood (4). Herein, this case report describes the presentation of two cases: one patient with encephalitis secondary to parainfluenza virus infection and another patient with RSV infection. These viruses were isolated in the respiratory tract and identified as the primary cause of neurological syndrome in the respective patients. Both children were managed in the pediatric intensive care unit (PICU) and had altered mental status, seizure, and hemodynamic instability.

## Case presentation

### Patient 1

A 5-year-old girl with no reported medical history presented with a persistent cough for 12 weeks, 3 days of fever, and lethargy in the last 24 h. She had three episodes of emesis and disorganized speech on the day of admission to the emergency department (ED). The patient was followed by a pediatric pulmonologist and allergist due to a cough. There was no history of previous hospitalizations.

**Figure 1:**
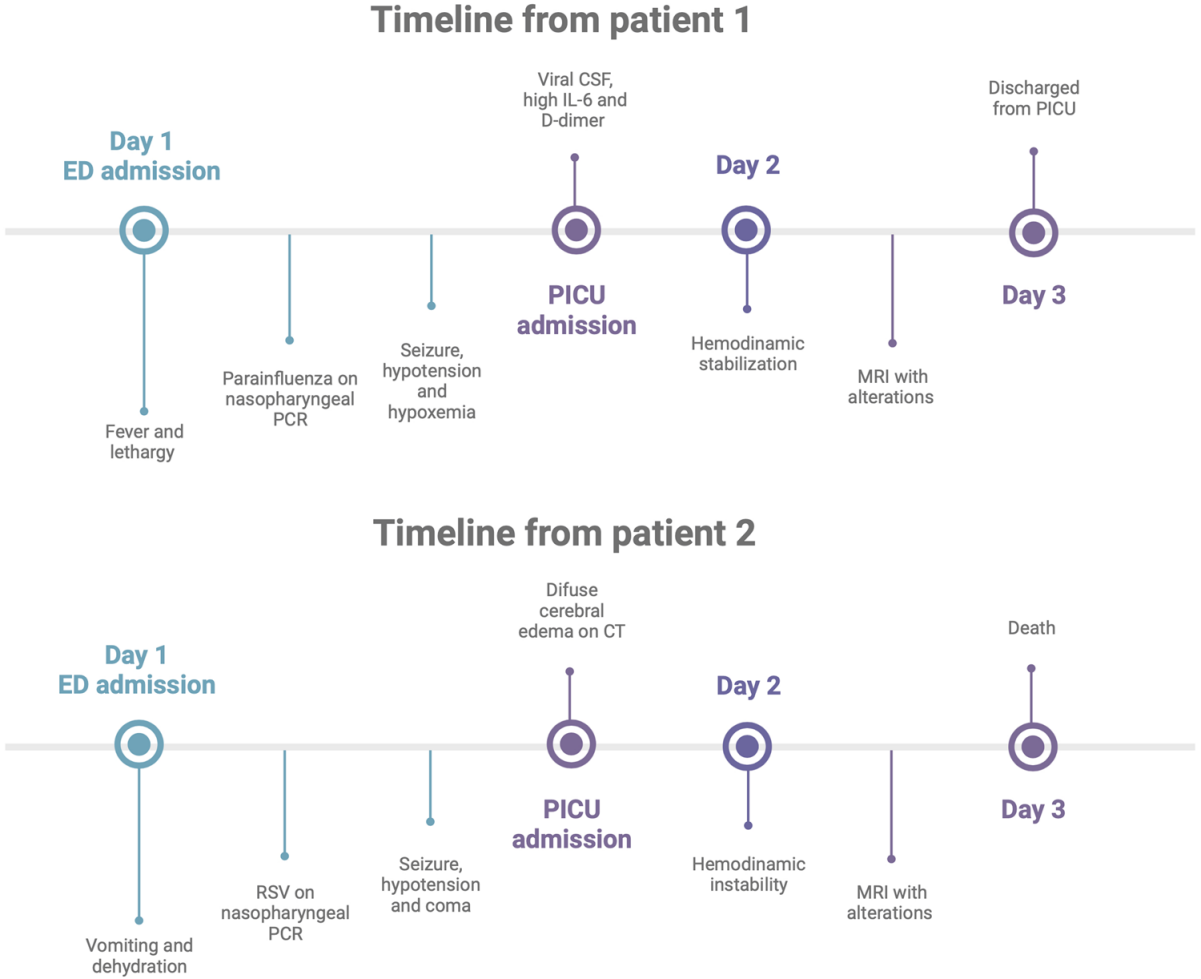
Patients' clinical event timeline: graphical representation.

In the ED, the patient was hypoxic, saturating 89% of room air, and received supplemental oxygen *via* a nasal cannula. She later had a single episode of prolonged generalized tonic–clonic seizure and rapidly developed hypotension and refractory hypoxia. She was treated with intravenous fluid bolus, epinephrine infusion, and mechanical ventilation and was started on empiric ceftriaxone and vancomycin. The patient was transferred to the PICU, where she was kept on mechanical ventilation and vasoactive drugs and sedated with dexmedetomidine (Figure 1).

**Figure 2:**
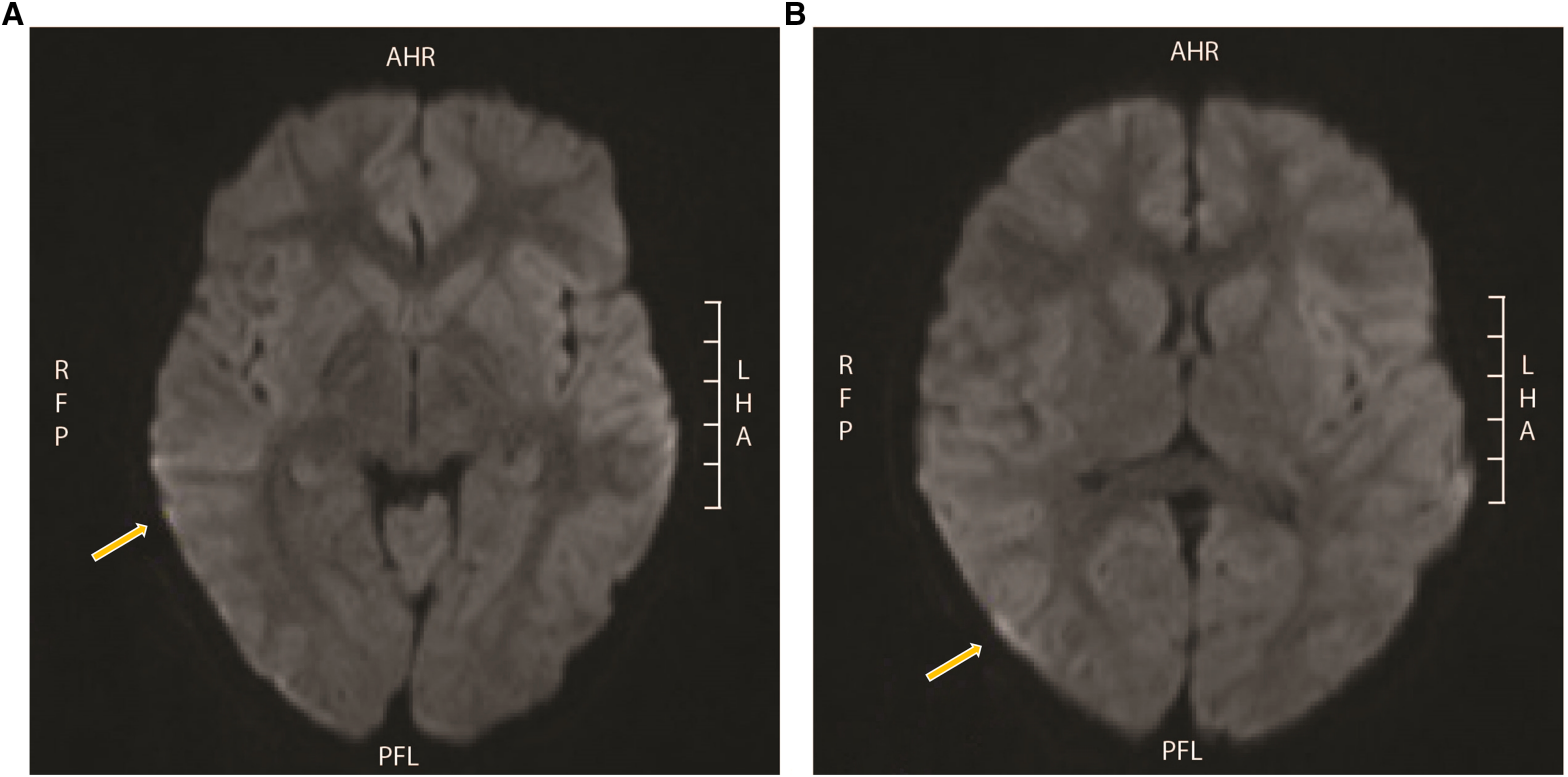
MRI from patient 1. Hypersignal on FLAIR within the sulci of the bilateral cerebral hemispheres, especially in the (**A**) posterior parietal and (**B**) occipital regions with minimal restricted diffusion in the extra-axial spaces of the bilateral temporal and occipital regions, and also increased linear enhancement in the sulci that is likely vascular and suggests the presence of hyperemia. MRI, magnetic resonance imaging; FLAIR, fluid-attenuated inversion recovery.

The echocardiogram and head computed tomography (CT) scan were normal. An electroencephalogram (EEG) showed diffuse slowing without seizure activity. MRI revealed hypersignal on the fluid-attenuated inversion recovery (FLAIR) sequence within the sulci of the bilateral cerebral hemispheres, especially in the posterior parietal and occipital regions (Figures 2A,B) with increased linear enhancement in the sulci, which suggests the presence of hyperemia and complete opacification of the bilateral mastoid air cells, middle ears, and petrous apices.

The lumbar puncture was compatible with viral infection: glucose 50 mmol/L, protein 25 mg/dl, 100 cells/µl with 75% lymphocytes. CSF Gram staining and culture were negative after 5 days, and CSF polymerase chain reaction (PCR) test was also negative for any organisms. The patient had a positive nasopharyngeal PCR for the human parainfluenza 3 virus. The workup for autoimmune encephalitis and multisystem inflammatory syndrome in children (MIS-C) linked to SARS CoV-2 (COVID-19) was also negative. This evaluation included anticardiolipin, dsDNA, ANA-Hep2 IgG, Beta-2 glyco1, C3 and C4, and ferritin. Initial C-reactive protein, IL-6, and D-dimer tests were elevated at 43 mg/dl (reference ≤ 1 mg/dl), 20.2 pg/ml (reference < 2 pg/ml), 3680 ng/ml (reference ≤ 499 ng/ml), respectively. The blood culture was negative after 5 days, and intravenous antibiotics were discontinued. She was treated with linezolid for mastoiditis.

The patient was extubated on the third day of hospitalization with a normal neurologic examination. MRI 5 days after admission showed resolution of the abnormal FLAIR signal in the bilateral posterior parietal and occipital sulcus. The patient was discharged from the hospital after 6 days of hospitalization with outpatient follow-up for pediatric infectious disease and pediatric neurology.

### Patient 2

A 2-year-old boy with a history of asthma, allergic rhinitis, and multiple food allergies presented to the ED after recurrent episodes of vomiting and dehydration. He was admitted to an inpatient pediatric service for intravenous fluids for a presumptive diagnosis of dehydration. On the second day of admission, he developed a single generalized seizure episode, followed by coma and hypotension, requiring vasoactive infusion and intubation. He was started on empiric broad-spectrum antibiotics and acyclovir and oseltamivir. A nasopharyngeal swab was positive for the RSV.

The patient was admitted to the PICU with a Glasgow coma scale (GCS) score of 3T, and an emergent CT scan of the head showed diffuse cerebral edema (Figure 1). He was treated with vasoactive infusions, mechanical ventilation, and continuous sedation with dexmedetomidine, fentanyl, and ketamine for airway protection and optimization of cerebral perfusion pressure. He received intravenous immunoglobulin and methylprednisolone for possible acute demyelinating encephalomyelitis. EEG showed severe diffuse suppression without seizure activity. MRI demonstrated extensive diffuse cortical edema with impeding herniation as manifested by effacement of basal cisterns, crowding of the cerebellar tonsils at the foramen magnum, and effacement of the fourth ventricle. An echocardiogram showed a moderate decrease in the left ventricle systolic function with an estimated ejection fraction of 30%.

On day 3 of hospitalization, the patient developed refractory cardiac arrest and was pronounced dead. Autoimmune, infectious encephalitis and COVID/MIS-C evaluations were negative. The family elected for autopsy, which revealed early evolving RSV infection and evidence of RSV infection with positive immunostaining in brain and lung tissues, with glial fibrillary acidic protein (GFAP) staining demonstrating clasmatodendrosis.

## Discussion

Encephalitis is caused by CNS inflammation secondary to infectious or noninfectious damage to the brain parenchyma (5, 6). In children, this disorder is characterized by lethargy (90%), lowered GCS (43%), unusual behavior (73%), CSF pleocytosis (66%), seizures (10%–45%), and coma (1, 2). The annual incidence of encephalitis worldwide is 3.5–7.3 cases per 100,000 people and is most common in the pediatric population (5, 7). There were approximately 7,000 pediatric hospitalizations for encephalitis in 2004–2013 (5, 8), and 40% of these patients were admitted to PICUs (8). Infections are the most frequent cause of encephalitis, representing 57% of all cases. Additional 25% of cases were associated with immune-mediated diseases, and 17% were idiopathic (2).

In the pediatric population, 1.2%–6.5% of the identified viral agents causing encephalitis have been associated with RSV and less than 1% with the *paramyxoviridae* family, including parainfluenza viruses (2, 3). Among children with RSV infection, 1%–7% of hospitalized patients will manifest neurologic symptoms (3). Although most RSV-infected patients do not develop neurological complications (3, 4), the high annual incidence contributes to a significant number of hospitalized children who develop CNS disturbances such as encephalitis (3, 9). Seizures due to RSV are associated with an increased risk of developing critical illness and admission to a PICU (3, 8).

**Figure 3:**
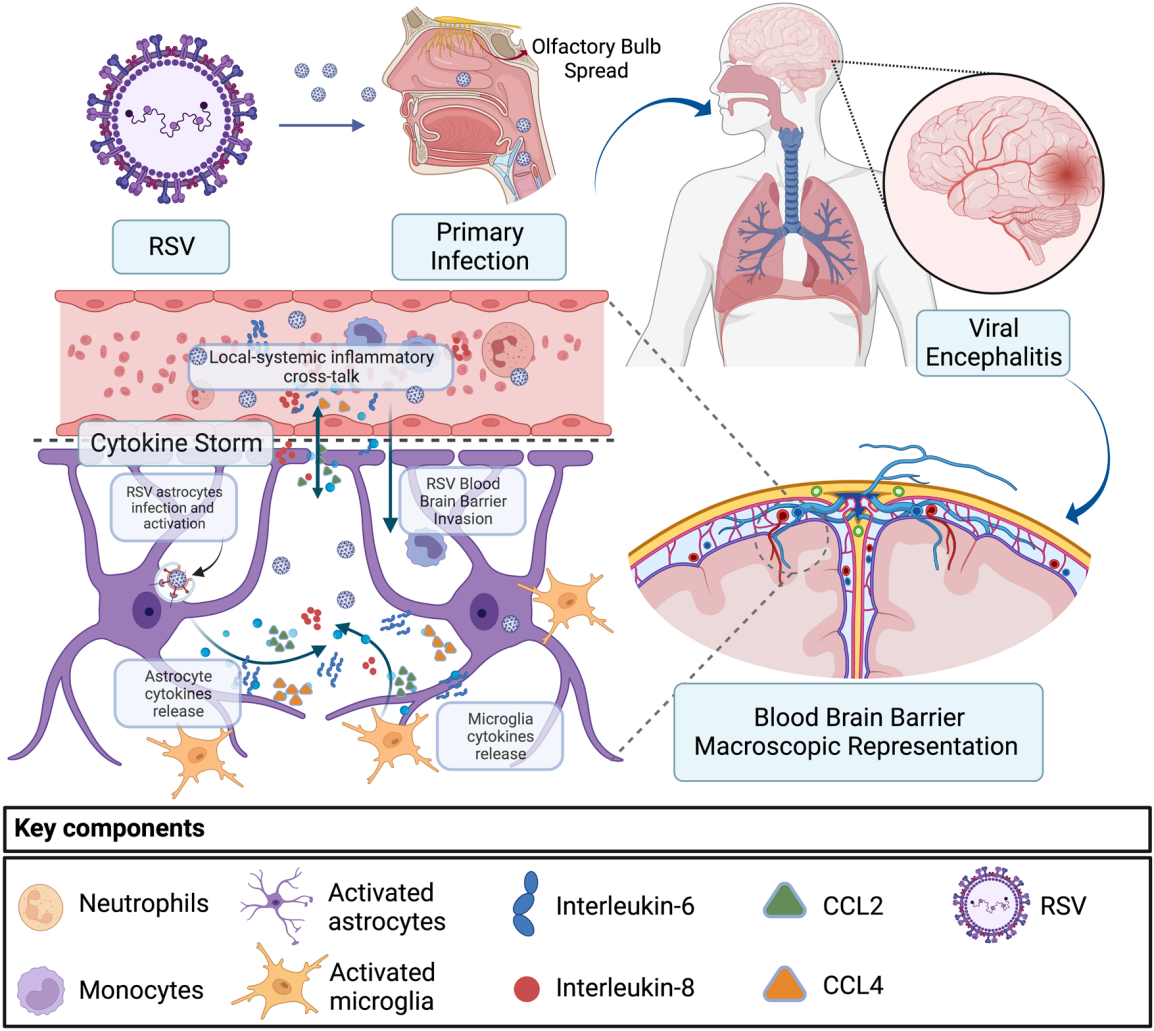
Encephalitis and cytokine storm secondary to RSV infection. RSV primary infection and spread from the respiratory tract to the CNS through the olfactory bulb or *via* the hematologic route. In the second scenario, RSV migrates through the blood–brain barrier infecting astrocytes and microglia, inducing upregulation of IL-6, IL-8, CCL2, and CCL4, which results in an eventual local-systemic inflammatory cross-talk that contributes to encephalitis and systemic inflammation. RSV, respiratory syncytial virus; CNS, central nervous system.

The pathophysiological mechanisms regarding how these viruses can propagate brain damage are not fully understood (4, 10). In both reported cases, we have common respiratory viruses, usually isolated in the respiratory tract causing neurological and systemic dysfunction. Possible routes of brain invasion by both RSV and parainfluenza viruses are through the olfactory sensory neurons or peripheral nerves and the bloodstream spread with CNS penetration after a viremia (4). With the detection of RSV RNA in the CSF, it is shown that this virus can reach the brain and can induce direct neuronal damage and cytokine production by astrocytes and microglia (Figure 3) (4, 10). In murine models, infection of brain cells by RSV can promote astrocyte activation and increase the production of cytokines and nitric oxide (4, 11). This correlates with reports of some patients with RSV encephalitis demonstrating elevated IL-6 levels in the CSF but not in the serum, pointing to a local production of cytokines in the neural system (4). Further, increased release of GFAP by astrocytes has been associated with neuroinflammation and cytokine upregulation (12).

Despite increased levels of cytokines in CSF, including IL-6, IL-8, CCL2, and CCL4, RSV could not be detected in cerebrospinal fluid samples (4, 13). This could be potentially explained by the migration of bloodstream inflammatory cells through the blood–brain barrier, leading to a severe inflammatory response in the brain as a result of a systemic and CNS proinflammatory cross-talk (10, 14). Cytokine storm linked with the onset and development of encephalitis is associated with local cytokine production in the neural system but may also drive systemic inflammation manifested with multiple organ failure, disseminated intravascular coagulation, and hemophagocytic syndrome (10, 11). Further studies are necessary to better understand the pathways and variables related to the RSV virus as the primary cause of encephalitis (4, 10).

Serial neurological examination and EEG are useful tools for detecting neurologic damage associated with encephalitis (6, 15). Approximately 83% of EEG in children with encephalitis display diffuse slowing and/or focal changes during the acute phase of encephalitis. MRI findings might be nonspecific and can demonstrate heterogeneous results even for the same etiology (3, 15). In patients with encephalitis due to respiratory virus infection, the most common MRI findings were bilateral basal ganglia and thalamic involvement (6, 16), and symmetrical lesions occurred more in this condition than other infectious agents (16).

Encephalitis mortality in children is 3% in the United States (5, 8), and morbidity due to learning difficulties, behavioral concerns, and motor dysfunction after viral encephalitis affects nearly 20% of hospitalized children (5, 10). Seizures with loss of consciousness, GCS < 13, abnormal EEG, and intensive care unit admission are associated with increased rates of neurological morbidity and death (2, 14). No specific drugs or therapies are available targeting RSV and parainfluenza in the setting of encephalitis. Treatment is supportive, and an early diagnosis is important to optimize the management of possible complications (17).

In summary, viral encephalitis is a common condition in the young population. These patients usually present mild symptoms that potentially progress to systemic inflammation and coma. Although cases of encephalitis secondary to respiratory viruses such as RSV and parainfluenza virus have been reported, the pathophysiological mechanisms of brain injury and systemic inflammatory response are still poorly understood. The low incidence of these atypical encephalitis viral agents can delay the suspicion and diagnosis. This article focuses on the importance of bringing awareness to the potential for cytokine storm and neurological damage development during encephalitis secondary to typical respiratory viruses.

## Data Availability

The original contributions presented in the study are included in the article/Supplementary Material; further inquiries can be directed to the corresponding author.
